# Supplementary material for: Convergent genomic and molecular features predict risk of metachronous metastasis in clear cell renal cell carcinoma
Source: Commun Med (Lond). 2026 Feb 25;6:205. doi: 10.1038/s43856-026-01436-6 (PMC13066385; doi:10.1038/s43856-026-01436-6)
Supplement: Supplementary file 3 — Description of Additional Supplementary files [file 43856_2026_1436_MOESM3_ESM.pdf]

Description of Additional Supplementary Files

Supplementary Data 1: Source data used to generate Figures 2-6.

Supplementary Data 2: Clinical information of the 192 TCGA ccRCC primary tumors

Supplementary Data 3: Differentially expressed genes in cancer cells of MM cohort versus IN cohort

Supplementary Data 4: Differentially expressed genes in stromal cells of MM cohort versus IN cohort
